# Genetic polymorphism of circumsporozoite protein of *Plasmodium falciparum* among Chinese migrant workers returning from Africa to Henan Province

**DOI:** 10.1186/s12936-022-04275-7

**Published:** 2022-08-27

**Authors:** Zhi-quan He, Qun-qun Zhang, Dan Wang, Ya-bo Hu, Rui-min Zhou, Dan Qian, Cheng-yun Yang, De-ling Lu, Su-hua Li, Ying Liu, Hong-wei Zhang

**Affiliations:** 1 Department of Parasite Disease Control and Prevention, Henan Center for Disease Control and Prevention, Zhengzhou, China; 2 Henan Key Laboratory of Pathogenic Microorganisms, No. 105 South Agricultural Road, Zhengdong New District, Zhengzhou, 450016 China; 3 Fengtai District Center for Disease Control and Prevention, Beijing, China; 4 College of Public Health, Zhengzhou University, Zhengzhou, China

**Keywords:** Imported malaria, *Plasmodium falciparum*, Circumparzoite protein, Genetic polymorphism, Henan Province, Africa

## Abstract

**Background:**

*Plasmodium falciparum* malaria is recognized as a major global public health problem. The malaria vaccine was important because the case fatality rate of falciparum malaria was high. *Plasmodium falciparum* circumsporozoite protein (PfCSP) is one of the potential vaccine candidates, but the genetic polymorphism of PfCSP raises concerns regarding the efficacy of the vaccine. This study aimed to investigate the genetic polymorphism of PfCSP and provide data for the improvement of PfCSP-based vaccine (RTS,S malaria vaccine).

**Methods:**

Blood samples were collected from 287 Chinese migrant workers who were infected with *P. falciparum* and returning from Africa to Henan Province during 2016–2018. The *Pfcsp* genes were analysed to estimate the genetic diversity of this parasite.

**Results:**

The results showed that there were two mutations at the N-terminus of imported *Pfcsp* in Henan Province, including insertion amino acids (58.71%, 118/201) and A → G (38.81%, 78/201). The number of repeats of tetrapeptide motifs (NANP/NVDP/NPNP/NVDA) in the central repeat region ranged mainly from 39 to 42 (97.51%, 196/201). A total of 14 nonsynonymous amino acid changes were found at the C-terminus. The average nucleotide difference (K) of imported *Pfcsp* in Henan Province was 5.719, and the haplotype diversity (Hd) was 0.964 ± 0.004. The estimated value of dN-dS was 0.047, indicating that the region may be affected by positive natural selection. The minimum number of recombination events (Rm) of imported *Pfcsp* in Henan Province was close to that in Africa. The analysis of genetic differentiation showed that there may be moderate differentiation between East Africa and North Africa (Fst = 0.06484), and the levels of differentiation in the other regions were very small (Fst < 0.05).

**Conclusions:**

The N-terminus of *Pfcsp* was relatively conserved, and the central repeat region and the Th2R and Th3R regions of the C-terminus were highly polymorphic. The gene polymorphism pattern among Chinese migrant workers returning from Africa to Henan Province was consistent with that in Africa. The geographical pattern of population differentiation and the evidence of natural selection and gene recombination suggested that the effect of polymorphism on the efficacy of PfCSP-based vaccines should be considered.

## Background

Malaria, caused by *Plasmodium* spp. infections, is one of the most significant life-threatening infectious diseases to humans worldwide. According to the World Health Organization (WHO) Malaria Report 2021, the total number of malaria deaths worldwide reached 627,000, equivalent to one death from malaria every minute in 2020. The incidence of malaria increased 69,000 in 2020 compared with that in 2019. However, affected by COVID-19, the diagnosis of malaria has declined, and the number of malaria deaths in sub-Saharan Africa has increased by 13%. [1]. *Plasmodium falciparum* is the most common parasite causing human malaria. It has the strongest pathogenicity and is also the main cause of severe malaria. Falciparum malaria patients with low immunity or untimely treatment easily develop severe malaria and even die [2, 3].

Malaria has historically been a major health problem in Henan Province [4]. In 2010, Henan Province launched an action plan to eliminate malaria, achieved no indigenous infection cases in 2012, and passed the assessment of malaria elimination in 2019 [5–7]. Although indigenous malaria transmission has been effectively controlled, the problem of imported malaria infection from abroad has become increasingly prominent. In recent years, with the development of global trade and the transnational economy, especially the increasing number of workers and businessmen in malaria-prone areas, such as Africa and Southeast Asia, overseas imported infections caused by population mobility have become the main source of malaria cases in Henan Province, which has brought new challenges to the overall elimination of malaria [8–10]. The vast majority of imported malaria cases come from Africa, and *P. falciparum* has become responsible for these infections.

Developing a malaria vaccine that provides durable protection against clinical disease and completely prevents infection will be critical for controlling and eliminating malaria. Anti-sporozoite vaccines, such as RTS,S, which target *P. falciparum* circumsporozoite protein (CSP) expressed on the surface of sporozoites, are leading malaria vaccine candidates undergoing phase III clinical trials in malaria-endemic areas [11, 12]. RTS,S malaria vaccine trials showed a significant effect in reducing the malaria incidence in many African countries including Ghana, Kenya, Mozambique, Gambia, Tanzania, and Gabon [13–15]. PfCSP is divided into three distinct regions: a highly variable central repeat region flanked by a conserved N-terminal region and a C-terminal nonrepeat region. The central repeat region, which has been recognized as a major target for antibody-mediated neutralization, is rich in Asn-Ala-Asn-Pro (NANP) tandem repeats and contains a small number of Asn-Vla-Asp-Pro (NVDP) motifs. The C-terminal nonrepeat region includes two polymorphism subregions, Th2R and Th3R, where T-cell epitopes have been identified [16–19]. PfCSP is predominantly distributed on the surface of sporozoites and has a molecular mass of approximately 58 kDa [20]. PfCSP has been found to show various genetic and antigenic polymorphisms in global parasites, which might obstruct or reduce the efficacy of vaccines [19, 21, 22]. The study of *Pfcsp* gene polymorphisms is an international research hotspot, but there have been few domestic research reports. This study aimed to determine the molecular characterization of falciparum malaria to produce a genetic characterization of *Pfcsp*, to understand the molecular evolution of the *Pfcsp* gene and to provide data for the improvement of PfCSP-based vaccines (including the RTS,S malaria vaccine).

## Methods

### Sample and data collection

*Plasmodium falciparum-*infected blood samples from patients, including finger-prick blood samples and venous blood samples, were collected. Blood samples (2 mL each) were collected from symptomatic patients before treatment. All of the patients came from Africa in 2016–2018. The samples were confirmed by PCR and microscopy examination. Samples were collected in Ethylene diamine tetraacetic acid (EDTA) tubes and transported to the Henan Province Center for Disease Control and Prevention (Henan CDC) the next day. The samples were stored at − 80 °C until laboratory analysis. A structured questionnaire was used to collect sociodemographic and clinical data from the subjects.

### DNA template preparation

*Plasmodium falciparum* genomic DNA was extracted from the blood samples using a QIAamp DNA Mini kit (Qiagen, Valencia, CA, USA) following the manufacturer’s instructions. TE buffer (10 Mm Tris–HCL, pH 8.0, 0.1 M EDTA) was used to dissolve the DNA and it was stored at − 20 °C until use. A 1.5% agarose gel stained with ethidium bromide was used to check the quality of the DNA and it was visualized with UV illumination.

### *Pfcsp* gene amplification and sequencing

A PCR amplification method was used to amplify the *Pfcsp* gene. The primers were as follows: PfCSP – F (5’-CGTGTAAAAATAAGTAGAAACCACG -3’), PfCSP – R (5’-TGTACAACTCAAACTAAGATGTGTTC -3’) [23]. Amplification reactions were performed in a 60 µL reaction volume containing 2 µL of DNA sample, 30 µL of a 2 × Go Taq Green Master Mix (Promega Inc., Madison, WI, USA), 3 µL of target primers, and 22 µL of ddH_2_O. PCR was performed with the following conditions: 94 ℃ for 1 min, followed by 35 cycles of 94 ℃ for 30 s, 50 ℃ for 30 s, and 72 ℃ for 2 min and a final extension of 72 ℃ for 5 min. Sequencing was conducted by Shanghai DNA Bio Technologies Co., Ltd. (Shanghai, China). All PCR products were analysed using 1.5% agarose gel electrophoresis and were then they were purified and sequenced by using an ABI 3730 × L automated sequencer. To ensure the accuracy of the sequencing, at least two clones for each isolate were sequenced.

### Statistical analysis

#### Sequence alignment and amino acid polymorphism analysis

Sequence alignment and analysis were carried out using Bio-Edit software. The amino acid sequences were compared with the 3D7 strain (XM_001351086) as a reference sequence. The sequences of the amplicons were aligned with published data from the 3D7 strain from the NCBI database by BLAST analysis.

#### Nucleotide polymorphism, natural selection, and gene recombination analysis

Nucleotide polymorphism, natural selection, and gene recombination were analysed using DnaSP 6.12.03 software [24]. For one of the indicators of nucleotide sequence polymorphism, nucleotide diversity (π) was calculated by the Jukes and Cantor method with a sliding window length of 10 bp and step size of 5 bp. The sliding window diagram was used to estimate the stepwise diversity between sequences. The values of segregating sites (S), number of haplotypes (H), and haplotype diversity (Hd) were calculated by DnaSP 6.12.03 software. To test the null hypothesis of *Pfcsp* neutrality, the rates of synonymous (dS) and nonsynonymous (dN) mutations were estimated and compared by MEGA 7.0.26 software [25].

Tajima's D test (α = 0.05) and Fu and Li's D and F test (α = 0.05) were used to evaluate the neutral theory of natural selection. Tajima's D and Fu and Li's D and F statistics were positive (D > 0, F > 0), indicating that it was a positive selection; A negative statistic (D < 0, F < 0) indicating that it was a negative selection [26].

R represents the occurrence of gene recombination. Ra is the recombination probability between adjacent nucleotides of each generation; Rb refers to the recombination estimation of the whole gene, that is, the effective population size; and Rm is the minimum number of reorganization events.

#### Population differentiation analysis

For population differentiation analysis, Arlequin 3.5.2.2 software and the R program were used to calculate the Fst index [27]. Fst was used to measure the degree of population differentiation, ranging from 0 to 1. If the Fst was 0 ~ 0.05, it indicated that the genetic differentiation between populations was very small, which cannot be considered; if the Fst was 0.05 ~ 0.15, it indicated that there was moderate genetic differentiation among populations; if the Fst was 0.15 ~ 0.25, it indicated that the genetic differentiation among populations was large; and if the Fst was more than 0.25, it indicated that there was great genetic differentiation among populations [28].

$$ F_{{{\text{ST}}}} = \frac{{H_{T} - H_{S} }}{{H_{T} }} $$

F_ST_ represents the inbreeding coefficient of subgroup (S) relative to the total population (T); H_T_ represents the expected frequency of heterozygotes in the total population under Harwin equilibrium; H_S_ represents the expected frequency of heterozygotes in the subgroup under Harwin equilibrium.

## Results

### Respondent characteristics

A total of 287 blood samples were collected from patients who were infected with *P. falciparum* returning from 27 countries of Africa to Henan Province during 2016–2018. The male: female ratio was 56.5:1 (282/5). The age ranged from 19 to 71 years old, of which the proportion of patients who were 18 to 55 years old was 98.61% (283/287). The imported patients all came from African countries, including countries in East Africa, West Africa, South Africa, North Africa, and Central Africa, which accounted for 7.66%, 40.07%, 28.23%, 1.05%, and 22.99% respectively. The positive rate of *Pfcsp* gene PCR amplification was 91.29% (262/287), and the size of the amplification product was 1100–1300 bp. After gene sequencing, 262 amplified positive products successfully obtained the full-length *Pfcsp* sequence. Ultimately, 201 full-length monoclonal *Pfcsp* sequences were analysed in this study, including 83 in West Africa, 57 in South Africa, 48 in Central Africa, 10 in East Africa, and 3 in North Africa, while 61 polyclonal *Pfcsp* sequences were excluded (Table 1).

**Table 1:** Basic information of falciparum malaria cases and PfCSP sequencing samples among Chinese migrant workers returning from Africa during 2016–2018

Characteristics	No. of cases	Composition ratio (%)	No. of successfully sequenced samples	Composition ratio (%)
Sex				
Male	282	98.26	197	98.01
Female	5	1.74	4	1.99
Age				
18–55	283	98.61	197	98.01
≥ 56	4	1.39	4	1.99
Source of infection				
West Africa				
Nigeria	45	15.68	32	15.92
Guinea	20	6.97	11	5.47
Ghana	15	5.23	15	7.46
Ivory Coast	10	3.48	7	3.48
Sierra leone	10	3.48	7	3.48
Liberia	9	3.13	7	3.48
Benin	2	0.70	2	0.99
Togo	2	0.70	0	0
Senegal	2	0.70	2	0.99
South Africa				
Angola	55	19.16	40	19.90
Zambia	13	4.53	9	4.48
Mozambique	8	2.79	4	1.99
Republic of South Africa	3	1.05	2	0.99
Malawi	1	0.35	1	0.50
Madagascar	1	0.35	1	0.50
Central Africa				
Congo	20	6.97	12	5.97
Democratic Republic of Congo	13	4.53	10	4.98
Cameroon	13	4.53	10	4.98
Equatorial Guinea	9	3.13	9	4.48
Chad	4	1.39	2	0.99
Central African Republic	4	1.39	3	1.50
Gabon	3	1.05	2	0.99
East Africa				
Tanzania	11	3.83	4	1.99
Uganda	9	3.13	5	2.49
South Sudan	1	0.35	1	0.50
Ethiopia	1	0.35	0	0
North Africa				
Sudan	3	1.05	3	1.50

### N-terminal gene polymorphism of *Pfcsp*

**Fig. 1:**
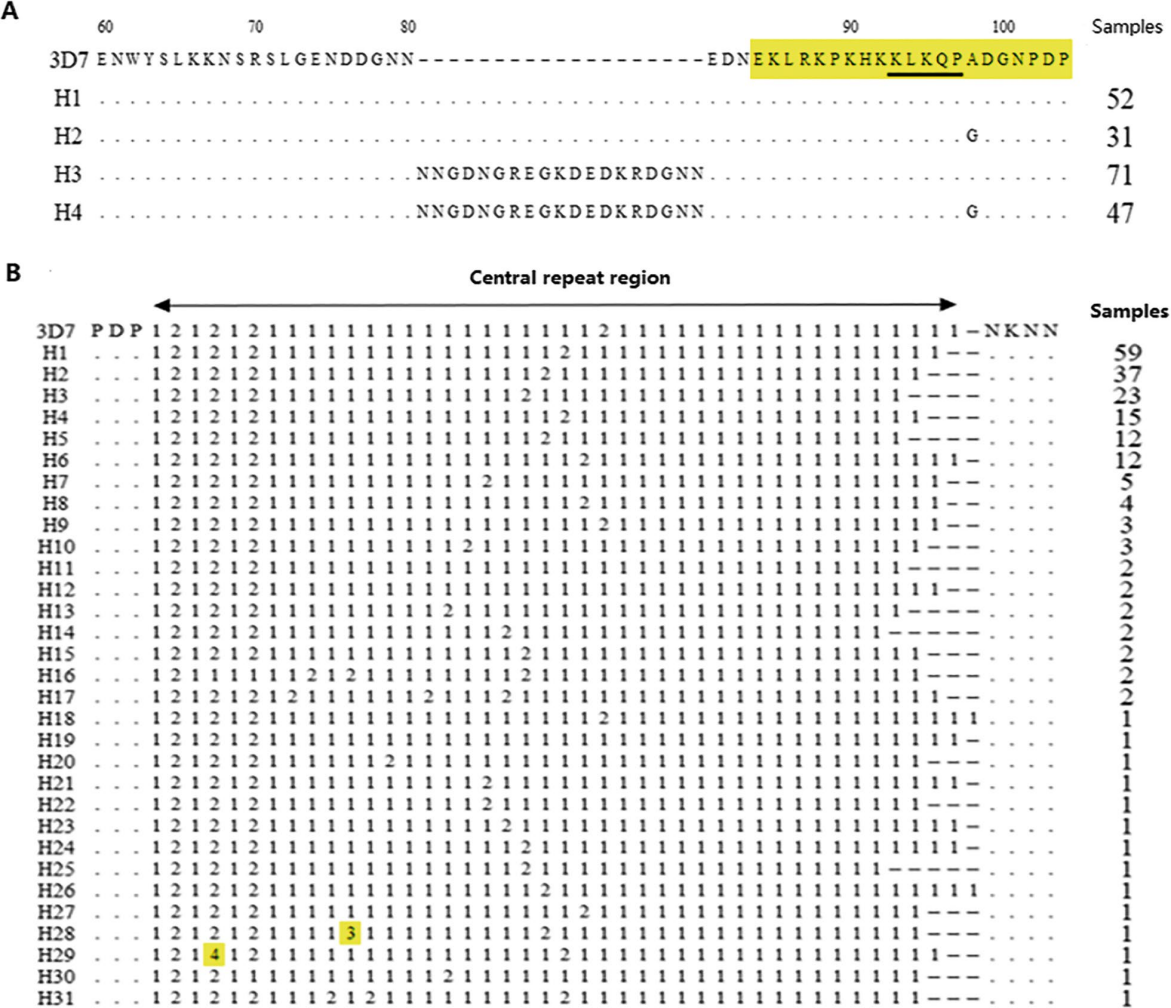
**A** Analysis of the N-terminal polymorphism of imported PfCSP in Henan Province. Dots represent the same residue as the 3D7 reference sequence. Dashes indicate intervals to maximize alignment. The yellow shaded area represents the predicted T-cell epitope area. The underlined conserved motif (KLKQP) was involved in the invasion of spores into mosquito salivary glands and bound to hepatocytes before invasion. The number of samples refers to the frequency of each haplotype. **B** Polymorphism characteristics of the central repeat region of imported PfCSP in Henan Province; 1: tetrapeptide motif NANP; 2: tetrapeptide motif NVDP; 3: tetrapeptide motif NPNP; 4: tetrapeptide motif NVDA; 1 and 2 represent known tetrapeptide repeats, and yellow shaded areas 3 and 4 represent newly discovered tetrapeptide motifs

The N-terminal nonrepeat region was relatively conserved, and there were four haplotypes. Compared to the 3D7 reference sequence (XM_001351086), H1 (25.87%, 52/201) was completely consistent with it. Two mutations occurred in H2, H3, and H4: one inserted a 19 amino acid fragment (NNGDNGREGKDEDKRDGNN) fragment after site 80 (58.71%, 118/201); the second was an A → G change at the 98^th^ base, i.e., A98G (38.81%, 78/201) (Fig. 1A).

### Central repeat region gene polymorphism of *Pfcsp*

As shown in Fig. 1B, thirty-one unique haplotypes were identified in imported PfCSP at the amino acid level. Haplotypes completely consistent with the 3D7 reference sequence were not found. Two repeat haplotypes encoding NPNP and NVDA were found in H28 and H29. Each haplotype of imported *Pfcsp* had a different number of tetrapeptide repeat motifs—NANP/NVDP/NPNP/NVDA. The number of repeats of tetrapeptide motifs ranged from 38 to 43, of which 40 (32.34%, 65/201) and 41 (37.81%, 76/201) had higher frequencies. These different numbers of repeats led to the polymorphism of *Pfcsp* in the central repeat region.

### C-terminal amino acid polymorphism of PfCSP

**Fig. 2:**
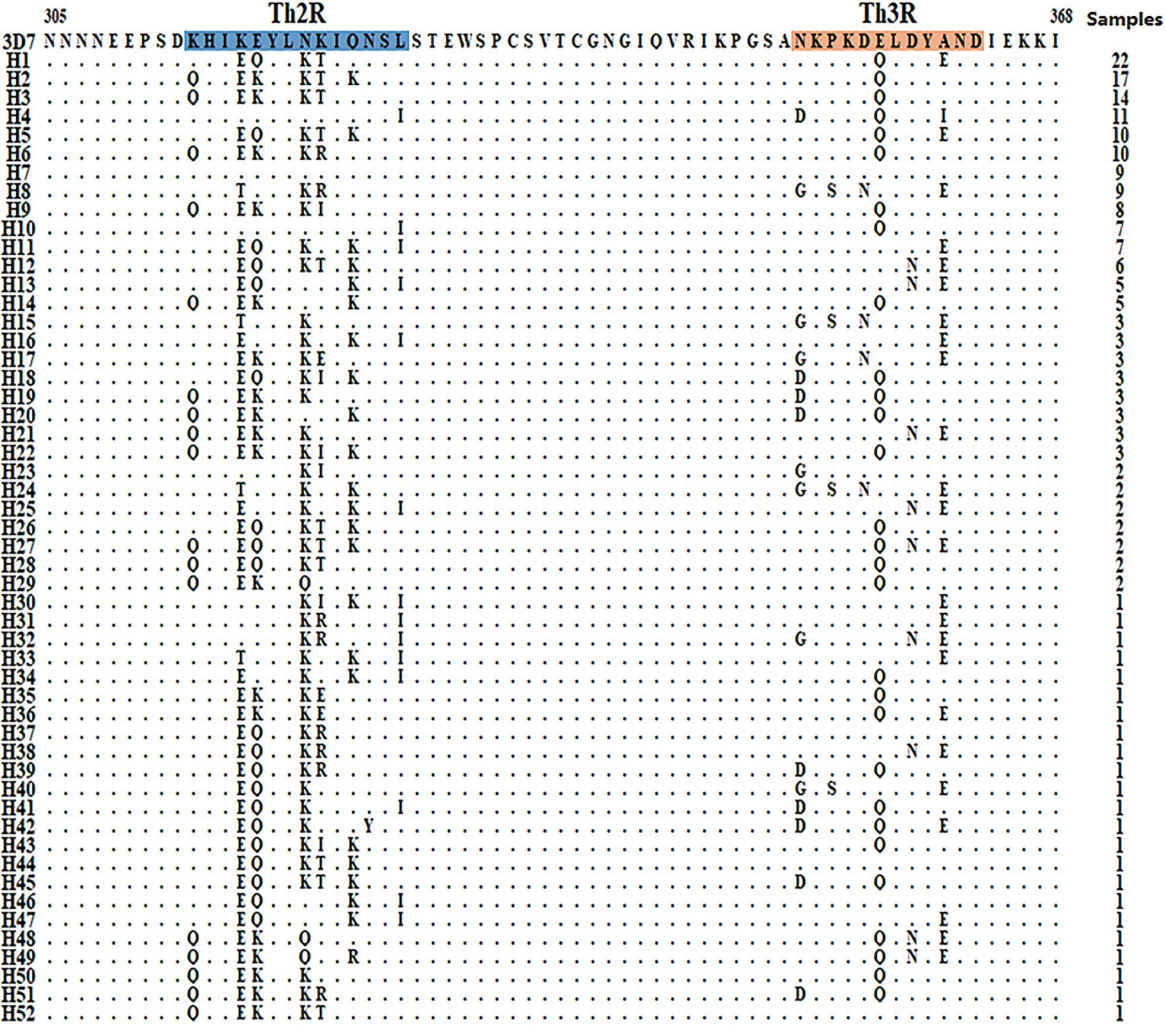
Analysis of C-terminal haplotypes of imported PfCSP in Henan Province; The dots represent the same residue as the 3D7 reference sequence. The blue shaded area represents the Th2R area. The orange shaded area represents the Th3R area. The number of samples refers to the frequency of each haplotype

A total of 52 different haplotypes (H1-H52) were identified in the C-terminal nonrepetitive region of imported PfCSP in Henan Province, of which H7 (4.48%, 9/201) was completely consistent with the 3D7 reference sequence. The Th2R and Th3R regions were highly polymorphic, and 14 nonsynonymous amino acid changes were found. A total of 8 of 14 were located in Th2R (^314^KHIKEYLNKIQNSL^327^), including K314Q, K317E/T, E318Q/K, N321K/Q, K322T/R/I/ E, Q324K/R, N325Y, L327/I. N352D/G, P354S, D356N, E357Q, D359N, while A361E/I were located in Th3R (^352^NKPKDELDYAND ^363^). Th2R and Th3R were identified as T-cell epitope regions (Fig. 2).

### C-terminal nucleotide polymorphism, natural selection, and gene recombination of *Pfcsp*

**Fig. 3:**
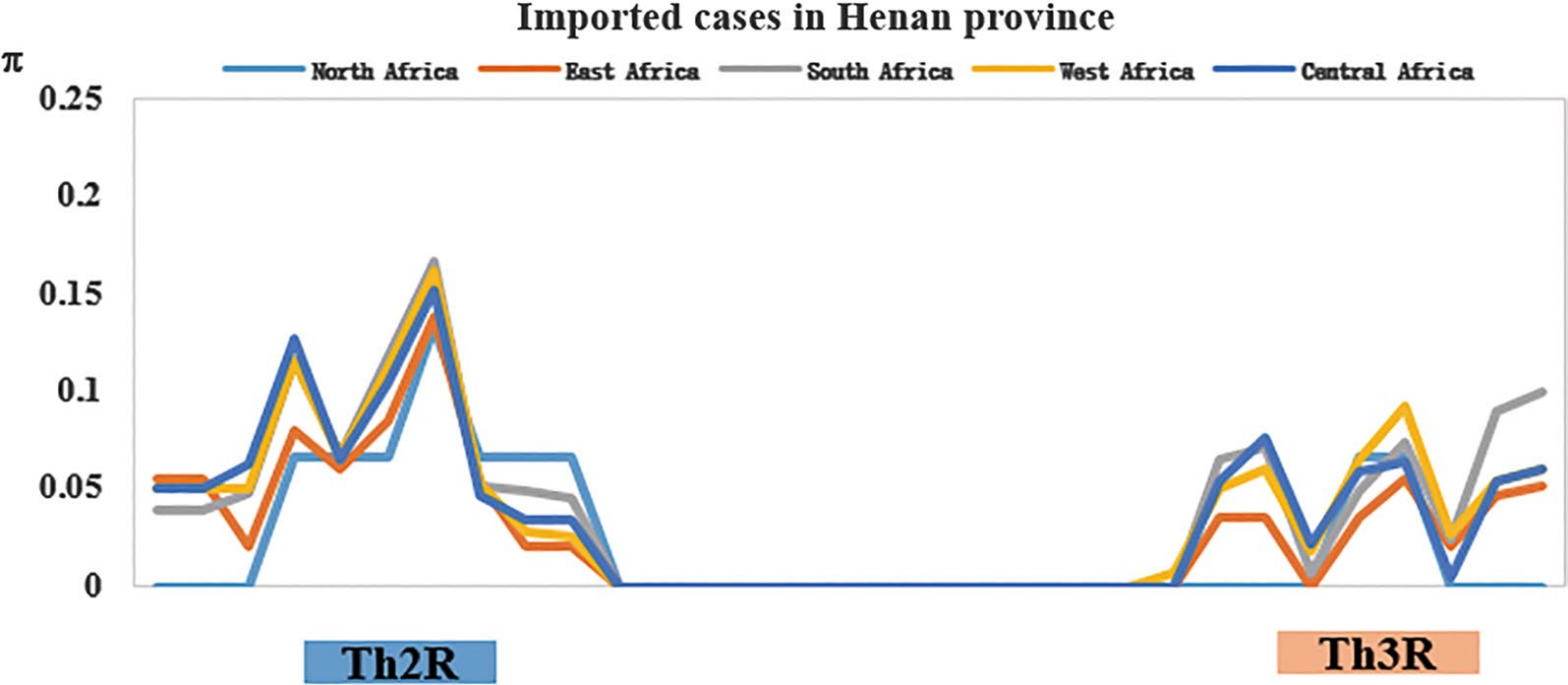
Analysis of the C-terminal nucleotide diversity of imported PfCSP in Henan Province. The nucleotide sequences of the C-terminal nonrepeat region (amino acid sites 311 ~ 363 fragments) of imported PfCSP in Henan Province were selected. The nucleotide diversity (π) was calculated by DnaSP software with a sliding window length of 10 bp and a step size of 5 bp

The nucleotide diversity (π) of the C-terminal nonrepeat region was analysed in the imported *Pfcsp* of Henan Province. The sliding window diagram showed that the T-cell epitope regions Th2R and Th3R had high nucleotide diversity, while the connecting region between Th2R and Th3R was highly conserved. The nucleotide diversity in the Th2R region was higher than that in the Th3R region in the imported PfCSP of Henan Province (Fig. 3).

For natural selection and gene recombination, the Henan Province imported *Pfcsp* and African native *Pfcsp* were evaluated. The average nucleotide difference (K) of the imported *Pfcsp* in Henan Province was 5.719, and the haplotype diversity (Hd) was 0.964 ± 0.004. The estimated value of dN-dS was 0.047, indicating that the region may be affected by positive natural selection. Although the neutral test results were not statistically significant, the statistics of Tajima's D value (D = 0.95250, *P* > 0.05), Fu and Li's D (D = 1.00540, *P* > 0.05) and F values (F = 1.18403, *P* > 0.05) were positive, indicating that the region may be experiencing positive selection. In addition, the Rm value of imported *Pfcsp* in Henan Province was close to that in Africa (Table 2).

**Table 2:** Natural selection test and recombination analysis of Henan Province imported PfCSP and African native PfCSP C-termini

Sample source	N		K	S	H	Hd ± SD	dN-dS	Tajima's D	Fu and Li's D	Fu and Li's F	Ra	Rb	Rm
Africa													
Congo	3		9.000	13	3	1.000 ± 0.272	0.076	–	–	–	—	—	—
Cameroon	9		4.556	12	7	0.944 ± 0.070	0.038	− 0.84239	− 0.68964	− 0.81319	0.1006	15.9	2
Kenya		18	5.843	18	13	0.928 ± 0.052	0.043	− 0.16823	− 0.13098	− 0.16414	0.1500	23.7	6
Ghana		34	5.062	18	19	0.930 ± 0.027	0.042	− 0.20329	0.46554	0.29096	0.1025	16.2	6
Gambia		44	5.966	18	21	0.951 ± 0.016	0.049	0.77207	1.34810	1.36435	0.1962	31.0	5
Tanzania		60	6.382	21	29	0.966 ± 0.009	0.053	0.60634	0.80724	0.87500	0.2013	31.8	6
Equatorial Guinea		96	4.906	21	33	0.948 ± 0.009	0.040	0.02365	− 0.40660	− 0.28991	0.1968	31.1	6
Total		264	5.566	27	60	0.959 ± 0.004	0.046	0.19441	− 0.70174	− 0.38625	0.1683	26.6	7
Cases from Africa back to Henan province													
North Africa	3		3.333	5	2	0.667 ± 0.314	0.027	–	–	–	—	—	—
East Africa	10		4.311	11	9	0.978 ± 0.054	0.035	− 0.28143	− 0.26257	− 0.30086	0.8861	140	2
Central Africa	48		5.561	17	23	0.942 ± 0.017	0.046	0.56577	0.29295	0.45903	0.1108	17.5	6
South Africa	57		6.056	18	30	0.963 ± 0.012	0.05	0.85180	1.37988	1.41794	0.3728	58.9	7
West Africa	83		5.692	20	34	0.964 ± 0.008	0.047	0.55706	0.32378	0.49098	0.1399	22.1	6
Total	201		5.719	21	60	0.964 ± 0.004	0.047	0.95250	1.00540	1.18403	0.1905	30.1	9

*N* Sample size, *SD* standard deviation

*P < 0.05; – The corresponding value cannot be calculated because there were few sequences (≤ 3)

### C-terminal population differentiation of *Pfcsp*

Through the analysis of genetic differentiation between populations, the genetic differentiation among imported *Pfcsp* populations in Henan Province showed that except that there may be moderate genetic differentiation between East Africa and North Africa (Fst = 0.06484), the genetic differentiation among other regions was very small (Fst < 0.05), which cannot be considered (Table 3).

**Table 3:** Population pairwise Fst index results

	East Africa	West Africa	South Africa	North Africa
West Africa	0.02407			
South Africa	0.01363	0.02290*		
North Africa	0.06486	0.01975	0.03297	
Central Africa	0.02497	0.00153	0.02355*	0.02045

Fst index was used to measure the degree of population differentiation, with a value of 0 ~ 1

*P < 0.05

## Discussion

The N-terminal region of PfCSP plays an important role in the process of sporozoite invasion into hepatocytes by mediating or promoting the interaction between sporozoites and host cells [29, 30]. The N-terminal genetic polymorphism in the imported PfCSP population in Henan Province was at a low level, and the N-terminal polymorphism pattern was consistent with the African PfCSP polymorphism pattern. This might also be related to the fact that the malaria cases imported from Henan Province were all from Africa. The above results were similar to those of previously reported studies [19–21]. According to the results of study by Huang et al., five variations were found in the *Pfcsp* N-terminal region of Bioko parasites including L5F, R70K, D82N, A98G, and a 57 bp insertion (encoding 19 amino acids: ^80^NNGDNGREGKDEDKRDGNN^81^) insertion. Two variations were found in the *Pfcsp* N-terminal region in this study. The study by Huang et al. demonstrated that A98G and 19 amino acid length insertions were universally popular while several novel mutations were found with low frequency [20]. Notably, none of the sequenced Sudanese isolates showed any insertions in the N-terminal region such as the 19 amino acid insertion (NNGDNGREGKDEDKRDGNN) that was found in the middle of the N-terminal region. This result was attributed to the sample sizes [31]. Larger sample size from other different regions and the selected regions of this study might provide different results if this insertion occurs by chance in the Sudanese *Pfcsp* gene [32]. The N-terminal region can be an attractive component of PfCSP-based vaccine due to the N-terminus of imported *Pfcsp* was relatively conserved.

The central repeat region of PfCSP played a key role in sporozoite formation and development [33]. The results of this study showed that the number of repeats of tetrapeptide motifs (NANP/NVDP/NPNP/NVDA) ranged from 38 to 43. Huang et al. reported that the numbers of repetitive sequences (NANP/NVDP) were mainly found as 40 and 41 in Bioko PfCSP [20]. Two repeat haplotypes encoding NPNP and NVDA were found in H28 and H29, and the result differed from the results of Lê et al. [19]: two novel repeat haplotypes, which encode NTNP and NANS motifs, were identified in two haplotypes (H3 and H9) of Myanmar PfCSP. Imported PfCSP in Henan Province had a high number of tetrapeptide repeats in the central repeat region, as 70.15% of *Pfcsp* had between 40 and 41 repeats. In addition, two novel tetrapeptide motifs NPNP and NVDA were found. Other tetrapeptide motif forms have been reported in the literature, including NVVP, NAKP, NAHP, NAIP, NVNP, NANL, NVAD, NADP, KANP, and SANP. It was unclear how these tetrapeptide motifs changed and how different positions affected the antibody response to CSP. The central repeat region is important in the PfCSP-based vaccine (RTS, S malaria vaccine). However, no studies indicated that the various number of tetrapeptide repeats can or may affect the effectiveness of the RTS, S malaria vaccine [34]. Therefore, the polymorphism in this region requires further in-depth study and analysis.

Abundant polymorphisms were found in the C-terminal analysis of PfCSP, especially in the thrombospondin type-I repeats (TSRs, small adhesive domains containing approximately 60 amino acid residues that mediate a broad range of biological interactions) region (including Th2R and Th3R), which confirmed T-cell immunogenic epitopes. The overall values of Hd (0.964 ± 0.004) in the C-terminal region of PfCSP were higher than those in previously reported studies [19, 21]. The genetic diversity in the C-terminal nonrepeat region among global PfCSP has been reported. The overall values for haplotype and nucleotide diversity for the PfCSP C-terminal region were higher in African PfCSP than in PfCSP from other continents, indicating that African PfCSP had a higher level of genetic diversity; the results of Zeeshan et al. was similar to this study [21]. The comparative analysis of the sliding window diagram of π in the C-terminal region showed that there were two peaks in the Th2R and Th3R regions, indicating that the genetic variation was mainly concentrated in these two regions. For natural selection of the C-terminal region, both Tajima's D and Fu and Li's D and F values were positive, indicating that the region may be experiencing positive selection, but these observations might be somewhat different from the study of Huang et al. [20]. Some previous studies revealed that the C-terminal region might be in a state of balanced selection to maintain or produce the genetic diversity of the global PfCSP population, and the value of Tajima's D in other regions were positive and highly polymorphic, which might be due to the balanced selection of this immunogenic epitope by host immune pressure [35–37]. The Rm value of imported PfCSP in Henan Province was close to that in Africa, possibly because these were people returning from Africa. Based on the study of Lê et al., these results indicate that high Rm values were predicted for African PfCSP, while lower levels of Rm were identified in PfCSP from other geographical areas, which may be due to the high polyclonal infection rate of this population and the subsequent cross-fertilization and active recombination of mosquitoes [19]. The RTS,S vaccine is composed of the C-terminal T-cell epitope, this region can be very important in terms of designing a specific vaccine.

The population differentiation analysis revealed that the genetic relationship between PfCSP in East Africa, West Africa, South Africa, and Central Africa was very close, and there was almost no differentiation, while North Africa and East Africa showed slight differentiation, which may be due to the small sample size of North Africa and the non-representativeness. Thus, the imported PfCSP did not consider geographical differences.

The limitation of this study was that there was no further study on the effects of the amino acid mutations on the structure or function of CSP to predict the effect of amino acid mutations on the efficacy of PfCSP-based vaccines.

## Conclusions

PfCSP is a main component of RTS,S, the most advanced malaria vaccine currently, but the genetic diversity in the *Pfcsp* gene among the different regions may affect the efficacy of the RTS, S malaria vaccine. In this study, the analysis of the genetic diversity of imported PfCSP in Henan Province indicated that N-terminus non-repeat region was relatively conserved, but the central repeat region and the Th2R and Th3R regions of the C-terminus were highly polymorphic. According to natural selection and gene recombination, the maintenance and production of genetic polymorphisms were speculated. The gene polymorphism pattern was consistent with that in Africa. These findings filled in missing data of imported PfCSP data in Henan Province and provided valuable information for the improvement of the PfCSP-based vaccines (including the RTS,S vaccine).

## Data Availability

The datasets used during the current study are available from the corresponding author on reasonable request.

## Abbreviations

PfCSP: *Plasmodium falciparum* Circumsporozoite protein
EDTA: Ethylene diamine tetraacetic acid
CDC: Center for Disease Control and Prevention
H: Haplotypes
Hd: Haplotype diversity

## References

[1] WHO. World Malaria Report 2021. Geneva: World Health Organization; 2021. https://www.who.int/teams/global-malaria-programme/reports/world-malaria-report-2021. Accessed 1 March 2022.

[2] Jie JJ, Liu JF, Liu DN, Liao Q, Chen J, Jiang WW (2019). Progress on virulence and gene promotion regulated by lncRNAs of *Plasmodium falciparum*. Adv Clin Med.

[3] Sagaki P, Thanachartwet V, Desakorn V, Sahassananda D, Chamnanchanunt S, Chierakul W (2013). Clinical factors for severity of *Plasmodium falciparum* malaria in hospitalized adults in Thailand. PLoS ONE.

[4] Liu Y, Zhang HW, Zhou RM, Yang CY, Qian D, Zhao YL (2014). First imported relapse case of *Plasmodium vivax* malaria and analysis of its origin by CSP sequencing in Henan Province. China Malar J.

[5] Guo WS, Zhao DY, Zhang HW, Lu DL, Liu Y, Qian D (2021). [Epidemiological characteristics of malaria in Henan Province from 1950 to 2019](in Chinese). Chin J Schisto Control.

[6] Yang CY, Lu DL, Zhou RM, Liu Y, Zhang HW, Zhao YL (2014). [Comparative analysis of malaria epidemic situation in Henan Province in 2011 and 2012](in Chinese). J Zhengzhou University.

[7] Zhang HW, Zhang QQ, Yang CY, Qian D, Lu DL, Zhao YL (2019). [Progress of malaria elimination and achievements of scientific researches in Henan province](in Chinese). Henan J Prev Med.

[8] Zhang QQ, Liu Y, Zhou RM, Yang CY, Qian D, Li SH (2020). [Diagnosis of imported malaria cases in Henan Province from 2015 to 2019](in Chinese). Chin J Schisto Control.

[9] Yang CY, Qian D, Lu DL, Liu Y, Zhou RM, Li SH (2020). [Epidemic status of malaria and progress of malaria elimination in Henan Province, 2018](in Chinese). Chin J Schisto Control.

[10] Feng J, Zhang L, Tu H, Zhou SS, Xia ZG (2021). [From elimination to post elimination: epidemic characteristics, challenges and strategies to prevent re transmission of imported malaria in China](in Chinese). Chin Trop Dis.

[11] Flores-Garcia Y, Wang LT, Park M, Asady B, Idris AH, Kisalu NK, et al. The P. falciparum CSP repeat region contains three distinct epitopes required for protection by antibodies in vivo. PLoS Pathog. 2021;17:e1010042. 10.1371/journal.ppat.1010042 PMC8601602 34748617

[12] Zavala F (2022). RTS, S: the first malaria vaccine. J Clin Invest.

[13] Asante KP, Abdulla S, Agnandji S, Lyimo J, Vekemans J, Soulanoudjingar S (2011). Safety and efficacy of the RTS, S/AS01E candidate malaria vaccine given with expanded-programme-on-immunisation vaccines: 19 month follow-up of a randomised, open-label, phase 2 trial. Lancet Infect Dis.

[14] Aponte JJ, Aide P, Renom M, Mandomando I, Bassat Q, Sacarlal J (2007). Safety of the RTS, S/AS02D candidate malaria vaccine in infants living in a highly endemic area of Mozambique: a double blind randomised controlled phase I/IIb trial. Lancet.

[15] Bojang KA, Milligan PJ, Pinder M, Vigneron L, Alloueche A, Kester KE (2001). Efficacy of RTS, S/AS02 malaria vaccine against *Plasmodium falciparum* infection in semi-immune adult men in The Gambia: a randomised trial. Lancet.

[16] Hughes AL (1991). Circumsporozoite protein genes of malaria parasites (*Plas-modium* spp.): Evidence for positive selection on immunogenic regions. Genetics.

[17] Waitumbi JN, Anyona SB, Hunja CW, Kifude CM, Polhemus ME, Walsh DS (2009). Impact of RTS, S/AS02(A) and RTS, S/AS01(B) on genotypes of *P. falciparum* in adults participating in a malaria vaccine clinical trial. PLoS ONE.

[18] Bailey JA, Mvalo T, Aragam N, Weiser M, Congdon S, Kamwendo D (2012). Use of massively parallel pyrosequencing to evaluate the diversity of and selection on *Plasmodium falciparum* CSP T-cell epitopes in Lilongwe. Malawi J Infect Dis.

[19] Lê HG, Kang JM, Moe M, Jun H, Thái TL, Lee J (2018). Genetic polymorphism and natural selection of circumsporozoite surface protein in *Plasmodium falciparum* field isolates from Myanmar. Malar J.

[20] Huang HY, Liang XY, Lin LY, Chen JT, Ehapo CS, Eyi UM (2020). Genetic polymorphism of *Plasmodium falciparum* circumsporozoite protein on Bioko Island, Equatorial Guinea and global comparative analysis. Malar J.

[21] Zeeshan M, Alam MT, Vinayak S, Bora H, Tyagi RK, Alam MS (2012). Genetic variation in the *Plasmodium falciparum* circumsporozoite protein in India and its relevance to RTS. S malaria vaccine PLoS One.

[22] Dobano C, Ubillos I, Jairoce C, Gyan B, Vidal M, Jimenez A (2019). RTS, S/ AS01E immunization increases antibody responses to vaccine-unrelated *Plasmodium falciparum* antigens associated with protection against clinical malaria in African children: a case–control study. BMC Med.

[23] Putaporntip C, Jongwutiwes S, Hughes AL (2009). Natural selection maintains a stable polymorphism at the circumsporozoite protein locus of *Plasmodium falciparum* in a low endemic area. Infect Genet Evol.

[24] Rozas J, Ferrer-Mata A, Sanchez-DelBarrio JC, Guirao-Rico S, Librado P, Ramos-Onsins SE (2017). DnaSP 6: DNA sequence polymorphism analysis of large data sets. Mol Biol Evol.

[25] Ina Y (1995). New methods for estimating the numbers of synonymous and nonsynonymous substitutions. J Mol Evol.

[26] Tajima F, Misawa K, Innan H (1998). The amount and pattern of DNA polymorphism under the neutral mutation hypothesis. Genetica.

[27] Excoffier L, Laval G, Schneider S. Arlequin (version 3.0): an integrated software package for population genetics data analysis. Evol Bioinform Online. 2007;1:47–50. PMC2658868 19325852

[28] Shriner D, Chen G, Adeyemo A, Rotimi CN (2016). Estimation of FST and the Impact of de novo Mutation. Hum Hered.

[29] Dundas K, Shears MJ, Sinnis P, Wright GJ (2019). Important extracellular interactions between *Plasmodium* sporozoites and host cells required for infection. Trends Parasitol.

[30] Ancsin JB, Kisilevsky R (2004). A binding site for highly sulfated heparan sulfate is identified in the N terminus of the circumsporozoite protein: significance for malarial sporozoite attachment to hepatocytes. J Biol Chem.

[31] Mohamed NS, Ali Albsheer MM, Abdelbagi H, Siddig EE, Mohamed MA, Ahmed AE (2019). Genetic polymorphism of the N-terminal region in circumsporozoite surface protein of *Plasmodium falciparum* field isolates from Sudan. Malar J.

[32] Mohamed NS, Abdelbagi H, Elsadig AR, Ahmed AE, Mohammed YO, Elssir LT (2021). Assessment of genetic diversity of *Plasmodium falciparum* circumsporozoite protein in Sudan: the RTS, S leading malaria vaccine candidate. Malar J.

[33] Ferguson DJ, Balaban AE, Patzewitz EM, Wall RJ, Hopp CS, Poulin B (2014). The repeat region of the circumsporozoite protein is critical for sporozoite formation and maturation in *Plasmodium*. PLoS ONE.

[34] Neafsey DE, Juraska M, Bedford T, Benkeser D, Valim C, Griggs A (2015). Genetic diversity and protective efficacy of the RTS, S/AS01 malaria vaccine. N Engl J Med.

[35] Amegashie EA, Amenga-Etego L, Adobor C, Ogoti P, Mbogo K, Amambua-Ngwa A (2020). Population genetic analysis of the *Plasmodium falciparum* circumsporozoite protein in two distinct ecological regions in Ghana. Malar J.

[36] Tetteh KK, Stewart LB, Ochola LI, Amambua-Ngwa A, Thomas AW, Marsh K (2009). Prospective identification of malaria parasite genes under balancing selection. PLoS ONE.

[37] Weedall GD, Conway DJ (2010). Detecting signatures of balancing selection to identify targets of anti-parasite immunity. Trends Parasitol.

